# Mediastinal Masses and Sternotomies: A Case Series

**DOI:** 10.7759/cureus.93693

**Published:** 2025-10-02

**Authors:** Joshua S Braganza, Michelle Davis, Caroline M Darch, Nadj L Pierre, Annis Ali

**Affiliations:** 1 Surgery, Wellspan York Hospital, York, USA; 2 Surgery, Drexel University College of Medicine, Philadelphia, USA; 3 Thoracic Surgery, Wellspan York Hospital, York, USA

**Keywords:** general thoracic surgery, mediastinum malignancy, paraganglioma, sternotomy, substernal goiter

## Abstract

Mediastinal masses or cervical masses with mediastinal extension can pose technical challenges when resection is chosen as the intervention of choice. In modern medicine, there are high volumes of minimally invasive approaches, and open surgery is becoming less common. Differentials for mediastinal masses include thymic tumors, thyroid masses, lymphomas, nerve tumors, and bronchogenic lesions, to name a few.

The authors describe two open approaches to mediastinal lesions, including a thyroid with mediastinal extension and a paraganglioma, which were both resected via the open approach through sternotomy and hemi-sternotomy. The first case was an 83-year-old female with a large thyroid goiter causing globus sensation and a workup consistent with a large thyroid goiter with mediastinal extension near the tracheo-esophageal groove, requiring sternotomy for resection. The second case was a 50-year-old male with a history of unprovoked deep vein thrombosis (DVT) incidentally found to have a mediastinal lesion between the left subclavian and left common carotid artery, which was growing in size. Resection was offered after a thorough workup. A robotic approach was offered and attempted; however, this was aborted in the setting of arterial bleeding. The patient ultimately required hemisternotomy and resection with postoperative complications of hydropneumothorax requiring tube thoracostomy and pulmonary embolism requiring therapeutic anticoagulation.

While both ultimately had successful resection, the older patient with sternotomy suffered no postoperative complications as compared to the younger male with the hemisternotomy. Nonetheless, this series highlights two cases where an open approach was required through sternotomy and hemisternotomy with successful resection of mediastinal masses.

## Introduction

The mediastinum is a central thoracic compartment bordered by the thoracic inlet superiorly, the diaphragm inferiorly, the sternum anteriorly, and the spine posteriorly [1]. The International Thymic Malignancy Interest Group (ITMIG) classification system, established in 2014, is the standard for localizing mediastinal masses, dividing the mediastinum into three compartments: prevascular (anterior), visceral (middle), and paravertebral (posterior) [2]. The anterior mediastinum is the most common site for primary mediastinal tumors, accounting for approximately 50% of all mediastinal masses [3]. The anterior compartment of the mediastinum is bordered ventrally by the undersurface of the sternum, dorsally by the pericardium, and laterally by the visceral pleura. The middle (or visceral) compartment spans from the thoracic inlet superiorly, the pericardium anteriorly, and extends to the anterior surface of the vertebrae posteriorly. The posterior compartment is defined anteriorly by the middle compartment and laterally by the costophrenic angle [1]. 

The classic differential diagnosis for anterior mediastinal masses follows the "4T" mnemonic: thymic tumors (thymoma, thymic carcinoma, thymic cysts), teratoma and other germ cell tumors, thyroid lesions (ectopic thyroid, goiter, thyroid carcinoma), and terrible lymphoma [4]. The differential diagnosis for middle mediastinal masses includes mediastinal cysts such as bronchogenic cysts, enterogenous cysts, neuroenteric cysts, pericardial cysts, and lymphangiomas. For posterior mediastinal masses, the differential diagnosis consists of neurogenic tumors (nerve sheath tumors, parasympathetic ganglion tumors, sympathetic chain tumors) and non-neurogenic tumors (chondrosarcoma, Ewing sarcoma, metastasis) [5].

The clinical presentation of mediastinal masses varies based on tumor size and location, with many patients being asymptomatic and diagnosed incidentally. Symptomatic cases often present with cough, chest pain, dyspnea, or superior vena cava (SVC) syndrome due to compression of adjacent structures [3]. Some tumors may also exhibit paraneoplastic syndromes such as myasthenia gravis in thymomas [1].

Diagnostic evaluation typically includes chest radiography, contrast-enhanced computed tomography (CECT), and occasionally, magnetic resonance imaging (MRI) or positron emission tomography (PET) to assess metabolic activity and disease extent [4,5]. 

Surgical management remains a mainstay of treatment for resectable mediastinal masses. Traditional approaches, such as median sternotomy, provide excellent exposure but are associated with significant morbidity. Minimally invasive techniques, including video-assisted thoracoscopic surgery (VATS), robotic-assisted thoracic surgery (RATS), and mini-sternotomy, have emerged as viable alternatives, offering reduced postoperative pain, shorter hospital stays, and comparable oncologic outcomes in appropriately selected patients [1,3].

## Case presentation

Case 1

An 83-year-old female, a smoker for 43 years, with a history of chronic obstructive pulmonary disease (COPD), not on home oxygen, and stage 3 chronic kidney disease (CKD III) presented to the otorhinolaryngology clinic with complaints of globus sensation, with imaging findings concerning for multiple enlarging thyroid nodules seen on ultrasound with substernal extension and lesions ranging from Thyroid Imaging Reporting and Data System (TIRADS) 3-5. The patient had compressive symptoms, especially with solid foods like bread. On physical exam, there was a soft, nontender, mobile 6x6 cm left thyroid mass and an ill-defined, non-tender fullness. Otherwise, there was no edema, erythema, or fluctuance, and the trachea was palpated to be midline with good landmarks. Additionally, there was no hoarseness. Given the detriment to her quality of life, she was interested in thyroidectomy.

**Figure 1:**
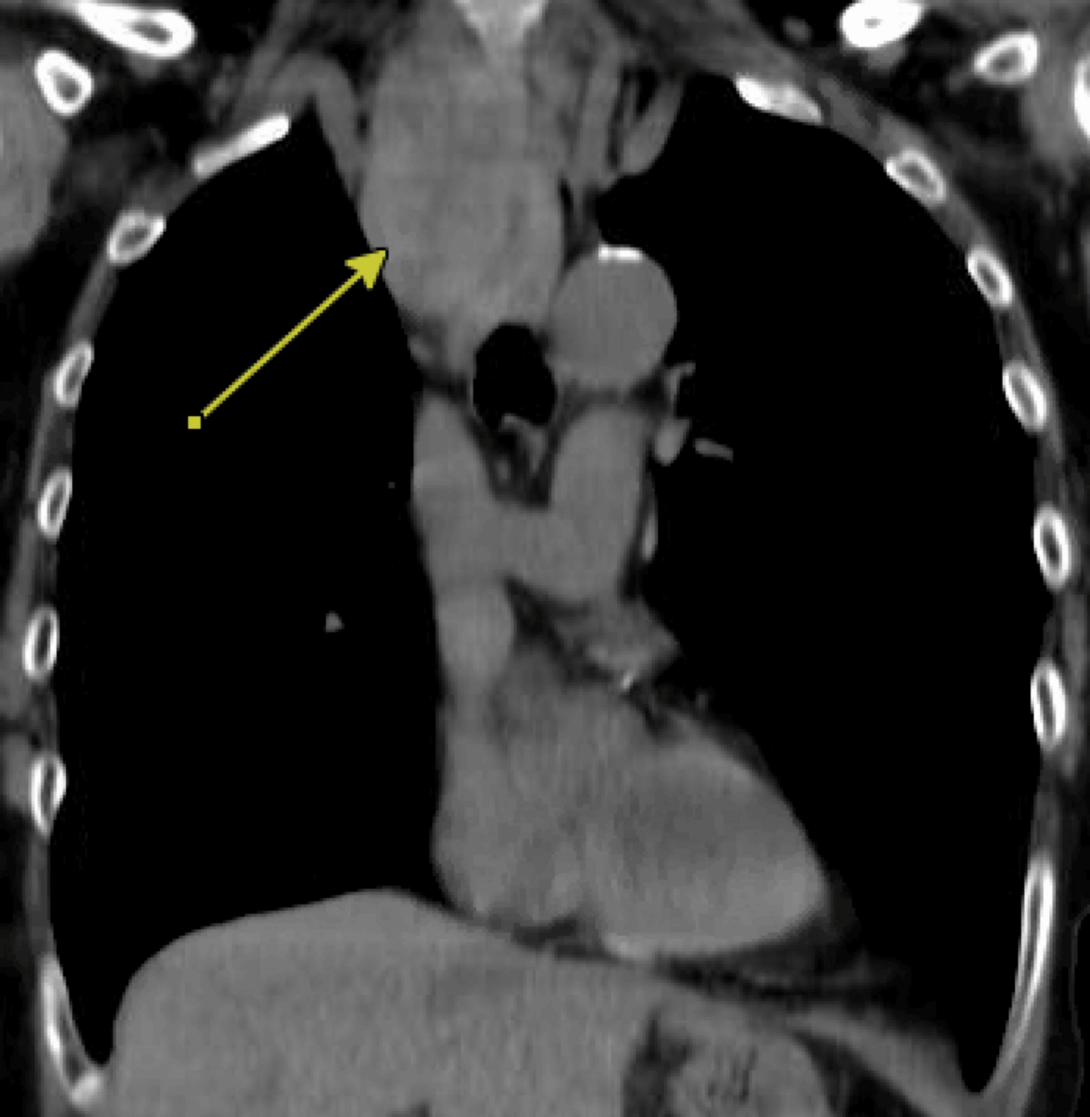
Case 1: CT scan of the chest Arrow pointing to the mediastinal goiter

Before referral to thoracic surgery, she underwent fine-needle aspiration (FNA) of not only her thyroid nodules, but also a left-sided parotid lesion. All thyroid nodules were noted to be Bethesda II, and the parotid lesion was noted to be a Warthin tumor. Given the substernal extension, a thoracic surgery referral was made, and a CT chest with IV contrast was obtained (Figure 1). The right thyroid lobe was noted to have retrosternal extension and to be between the trachea and esophagus, with extension beyond the carina. Pulmonary function tests were also obtained, given a significant smoking history and a COPD history, in light of possible plans for video-assisted thoracoscopic surgery (VATS) initially. Ultimately, the final plan was to proceed with hemisternotomy for thoracic exposure to allow for total thyroidectomy, which was made in conjunction with otorhinolaryngology.

The surgery began with a transverse incision, and skin and platysmal flaps were raised. Strap muscles were dissected out and retracted laterally. The left lobe was initially dissected, and the superior thyroid artery was divided, taking care not to injure the superior laryngeal nerve. The middle thyroid vein was divided next, and the recurrent laryngeal nerve was then identified, confirmed with the nerve stimulator, and protected. Medial dissection of the left thyroid lobe was continued until Berry’s ligament was divided. The left lobe was divided from the right and sent for permanent pathology.

Attention was shifted to the right thyroid lobe, which began similarly to the left lobe, with the division of the superior thyroid artery, taking care not to injure the superior laryngeal nerve. The middle thyroid vein was divided next, and dissection continued inferiorly until no further blunt dissection could be performed. A sternotomy was performed connecting to the cervical incision. The sternal saw was used to divide the manubrium and sternum in a cephalocaudal direction, and retractors were placed to facilitate anterior mediastinal exposure. The thyroid lobe appeared to extend between the trachea and esophagus. A nasogastric tube was placed to assist with identification of the esophagus, and dissection continued, taking care not to injure the right recurrent laryngeal nerve. The mass was delivered externally and examined. It was felt that the entirety of the mass had been resected, and wound closure was performed. The sternal edges were covered with vancomycin paste and re-approximated with sternal wires. Before skin closure, a 10-Fr drain was placed deep to the platysma extending into the superior mediastinum. A layered closure was performed in standard fashion.

The patient was admitted for three days postoperatively, with close monitoring on the intermediate care floor, and was discharged after pain had been adequately controlled, diet tolerated, labs stable, and evaluation by physical and occupational therapy had occurred. Her surgical drain was removed before discharge. She was discharged on Synthroid and a course of Tums and calcitriol, which was weaned by ENT.

On her follow-up 4 days post-discharge and once more 10 days later, she was doing well. Her pathology results were discussed with her and noted to be benign adenomatoid nodules. On the most recent follow-up three months later, her only complaints were musculoskeletal in her extremities and some adjustment disorder in response to personal stress. Her Synthroid dose has since been decreased, and she seems to be doing well.

Case 2

**Figure 2:**
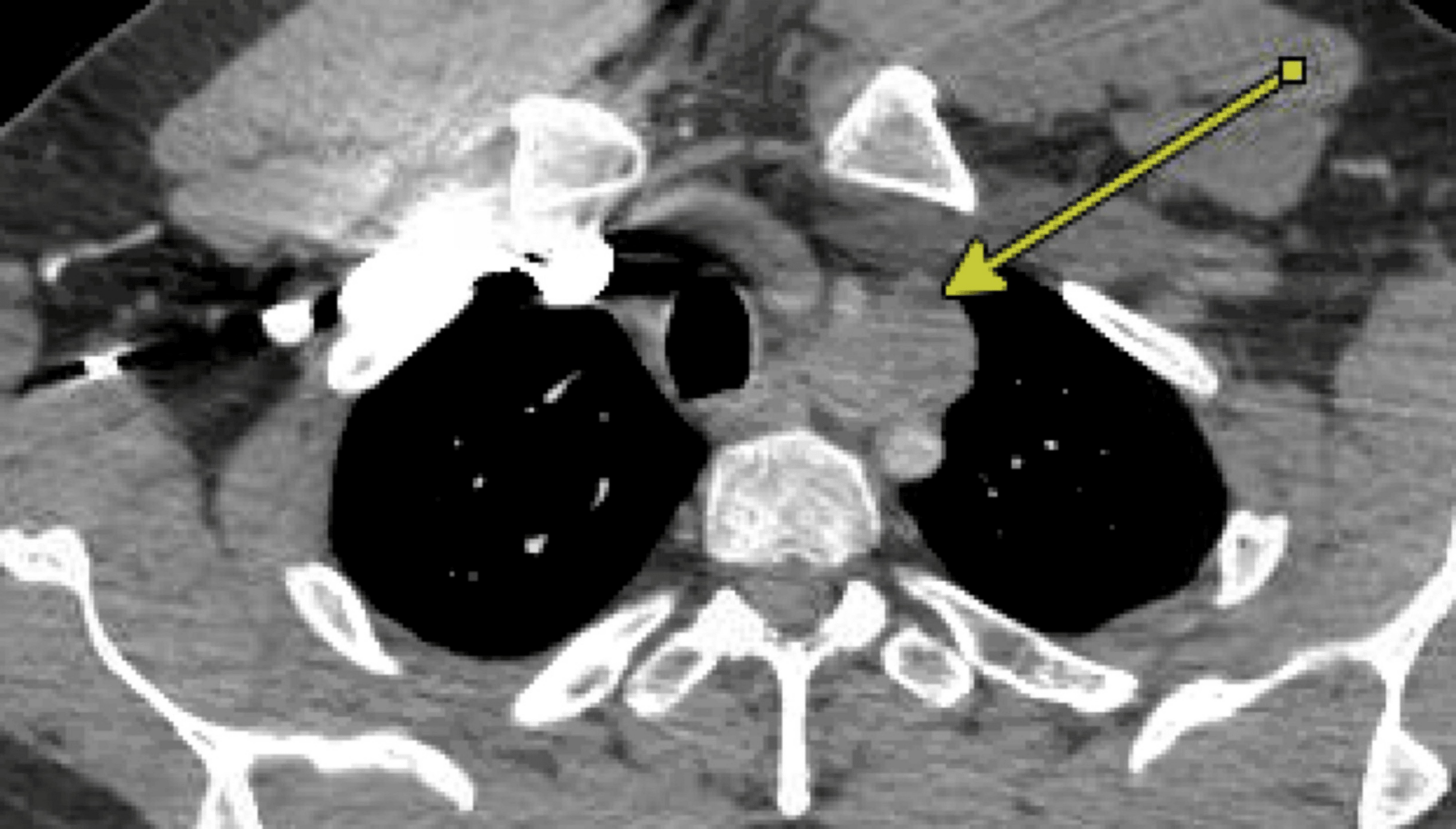
Case 2: CT scan of the chest Arrow pointing at the lesion near the aorta

A 50-year-old smoker for 12 years with a history of COPD, unprovoked right popliteal deep vein thrombosis, and coronary artery disease with prior diagnostic left heart catheterisation presented to the thoracic surgery clinic after incidental findings of a superior mediastinal tumor located between the left subclavian and left common carotid artery on a CT scan performed for evaluation of possible pulmonary embolism. The mass was noted to be 3.1 x 2.9 x 2.8 cm in size on CT scan (Figure 2). After a thorough history and physical exam were performed, a decision was made to obtain a carotid duplex to assess for any compressive effects, as well as a PET scan to further assist with differential diagnosis. PET scan revealed mild fluorodeoxyglucose (FDG) avidity and no other FDG-avid lesions. Left robotic-assisted thoracoscopic open resection of the tumour was offered, given the growth of the tumor from 2018 to 2024, the potential to continue to grow, and the proximity of the left common carotid and subclavian artery.

After informed consent was obtained, the patient was brought to the operating room and positioned and draped in standard fashion. Intubation was performed with a double-lumen endotracheal tube. After confirming that the left lung was deflated, an 8 mm port was inserted via the cutdown technique in the auscultatory triangle at the sixth intercostal for entry, and no injury was noted. Three additional 8 mm ports were placed in the eighth intercostal space to allow for docking of the robot. There were no signs of metastatic implants. The mass was identified and noted to be well-circumscribed between the left common carotid and left subclavian artery. Using a bipolar device, the pleura was opened, and the phrenic nerve was identified and protected. Of note, there was an additional nerve running anterior to the mass; however, it was not felt to be phrenic, vagus, or recurrent laryngeal. We began the dissection of the mass away from the great vessels, including the aortic arch; however, there was significant bleeding, requiring manual pressure and application of Surgicel (Johnson & Johnson MedTech, New Brunswick, NJ, USA). Attempts were made to continue dissection; however, arterial bleeding was encountered and not controlled with pressure and application of the Surgicel topical hemostatic agent. An EVARREST hemostatic patch (Johnson & Johnson MedTech) was placed, and hemostasis was achieved. The mass was extremely hypervascular, and there was concern that there may be an arteriovenous malformation (AVM). The decision was made to undock and have an additional discussion regarding surgical planning with the patient.

At follow-up in the office, CT angiography of the neck with venous phases was obtained and did not reveal an AVM; however, there was concern that this was an ectopic thyroid gland, and otorhinolaryngology referral was placed for further evaluation. MRI and US of the neck were performed, and a lymph node and a TIRADS 3 nodule were identified. US-guided FNA was performed on both the thyroid nodule and lymph node, which was noted to be benign. Furthermore, it was felt as though the mediastinal mass was not an ectopic thyroid, and a decision was made to proceed with hemisternotomy for open resection.

After informed consent was obtained, the patient was brought to the operating room and positioned, prepped, and draped in standard fashion. A hemisternotomy from the manubrium down to the level of the third intercostal space. Skin incision was extended toward the left sternocleidomastoid, and the strap muscles were divided to improve exposure. Retractors were placed, and the innominate vein was dissected out and retracted using a vessel loop. Dissection continued along the great vessels until the mass was fully exposed and noted to be in close proximity to the left common carotid artery and left subclavian artery. Nerves were identified and protected. A small specimen was sharply excised and sent for permanent pathology. Using a combination of the Ligasure vessel sealer (Medtronic, Minneapolis, MN, USA) and sharp dissection, the mass was successfully dissected away from the great vessels and aortic arch. The specimen was sent for pathology, and hemostasis was assured. The sternal edges were coated with antibiotic paste, and the sternum was reapproximated using sternal wire. The skin and subcutaneous tissues were closed in standard fashion. The patient was observed for two days postoperatively and was weaned from oxygen, tolerating diet, ambulatory, and had adequate pain control. 

**Figure 3:**
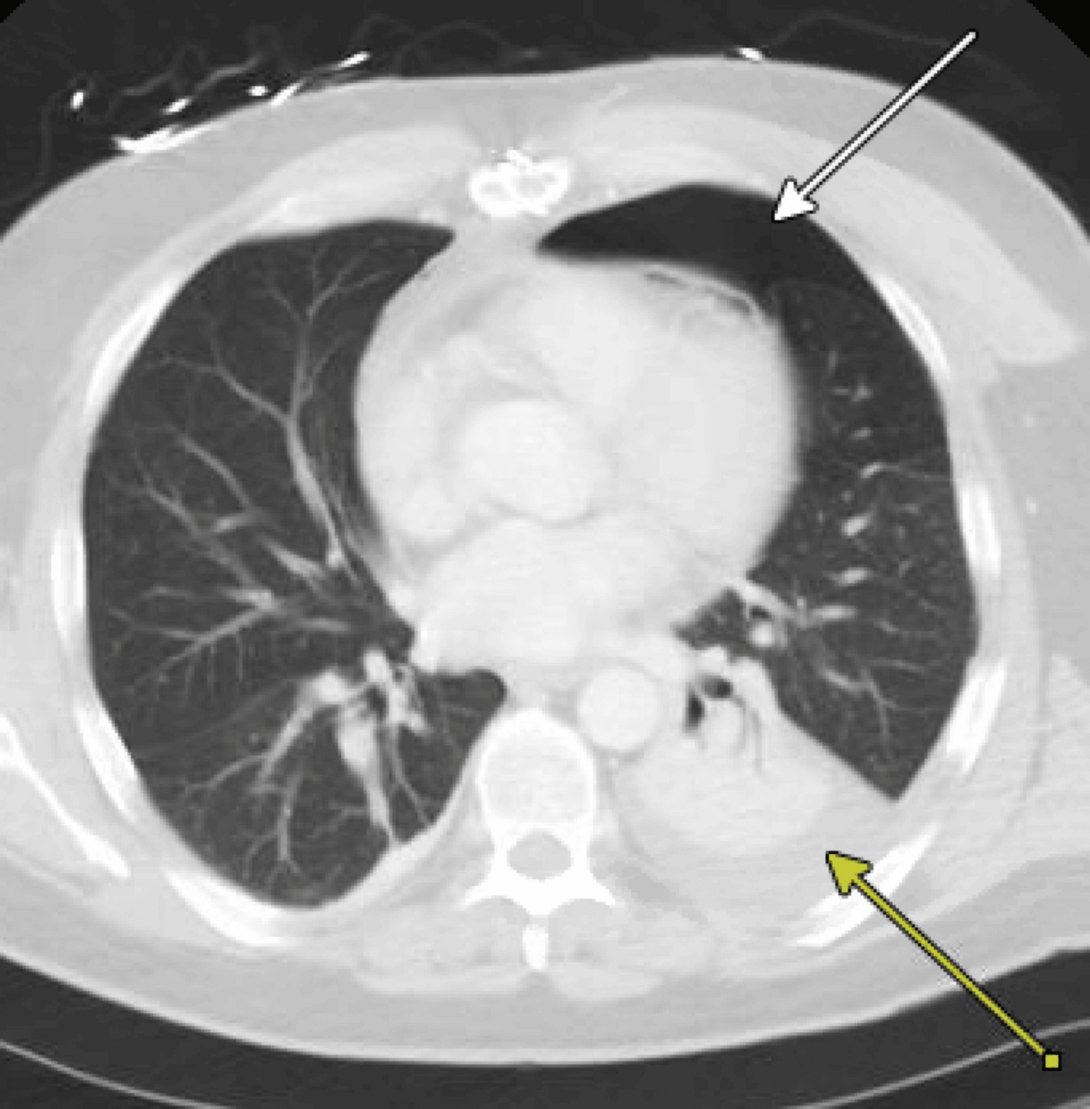
Case 2: CT scan of the chest post-discharge Left-sided hydropneumothorax with labels on the pneumothorax (white arrow) and hemothorax (yellow arrow)

Three days post-discharge, the patient presented to an outside hospital with shortness of breath and hypoxia requiring 15L of supplemental oxygen via a high-flow nasal cannula with imaging findings concerning for hydropneumothorax (Figure 3), requiring placement of a chest tube and ICU admission. The chest tube was eventually removed in five days with resolution of symptoms and hydropneumothorax. 

**Figure 4:**
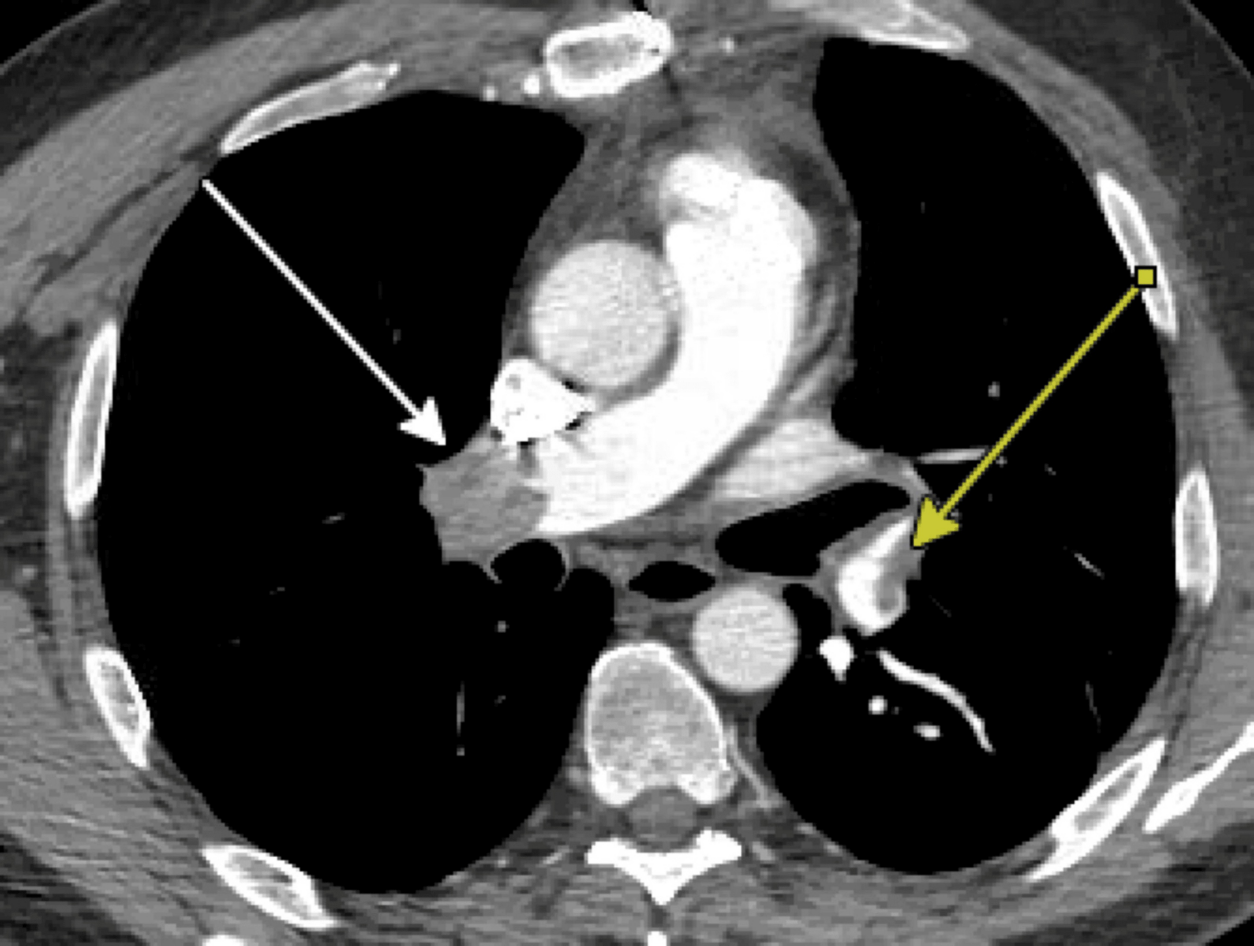
Case 2: CT angiography (CTA) pulmonary embolism (PE) revealing bilateral pulmonary embolisms Bilateral pulmonary embolisms marked with arrows

Two days post-discharge from his hospitalization in the ICU, he re-presented with shortness of breath once more and was found to have bilateral distal main pulmonary artery embolisms requiring anticoagulation, as seen on CT scan (Figure 4). A transthoracic echocardiogram was performed without evidence of right heart strain, although troponins were slightly elevated. The patient was treated with medical management after review of his case by the pulmonary embolism response team and was eventually transitioned off the heparin drip and onto an oral anticoagulant. He was weaned off oxygen and discharged, saturating well on room air on hospital day 5 from this admission.

On follow-up in the office with the thoracic surgery service, pathology results were discussed, and it was noted to be a paraganglioma. He was seen again one month postoperatively and was doing well without sequelae of his pulmonary embolism or hemisternotomy. He was cleared to return to work.

## Discussion

Both cases presented superior mediastinal lesions of different etiologies. While the approach of minimally invasive surgery is desirable, these two cases present two situations where it was not necessarily feasible. The first case highlights the substernal extension of a large right thyroid lobe extending to the tracheo-esophageal groove, requiring a full sternotomy for adequate mediastinal exposure. The second case presents a paraganglioma in close proximity to the left subclavian and common carotid arteries, which was hypervascular and not amenable to robotic-assisted resection. While both patients made it through surgery, the postoperative course was different, with the younger and seemingly healthier patient having more complications, including bilateral main branch pulmonary embolisms requiring anticoagulation and hydropneumothorax requiring chest tube placement and ICU admission. Although both complications are undesirable, the patient did have some risk factors for the pulmonary embolisms, given his history of prior unprovoked DVT. The remainder of the discussion explores the surgical approaches and challenges encountered. 

Substernal goiters are diagnosed more frequently in patients 50 years old or older and predominantly in females [6,7]. The etiology of the substernal goiter is similar to that of a cervical goiter, as the blood supply of the thyroid remains from the inferior and superior thyroid arteries. Worldwide, the most common cause of goiter is iodine deficiency [8]. Other etiologies include autoimmune and thyroid affecting drugs (i.e., Amiodarone, lithium) [8]. In a study performed by Sormaz et al. in 2018 to predict the need for extracervical approach for retrosternal thyroid lesions, a pre-operative volumetric analysis done with CT was used, and the craniocaudal length of the thyroid mass below the thoracic inlet of ≥66 mm or a volume of the mediastinal portion ≥162 cm3 were significantly associated with an extra-cervical approach (p=0.0001) [7]. In our case, the mass was clearly more than 66 mm below the thoracic inlet and, therefore, raised a high suspicion for the need for an extracervical approach. Approaches described in the literature include hemi-sternotomy, sternotomy, and thoracotomy [9]. Additionally, for a full sternotomy, a retrospective review was performed at the University of Wisconsin in 2016, with results showing that the need for full sternotomy typically occurs when the thyroid lesion extends below the level of the aortic arch, which was found in our patient [10].

Mediastinal paragangliomas account for 0.3% of mediastinal masses and are more commonly found in the posterior mediastinum [11,12]. They are slow-growing, hypervascular tumors, but are associated with high morbidity and mortality due to local invasion to the heart, great vessels, esophagus, and trachea [13]. These tumors can be identified on imaging studies, often located at the bifurcation of the great vessels [14]. In this case, the approach to the mass was guided by the previous robotic approach, which demonstrated the hypervascular nature and proximity to the great vessels; thus, a hemisternotomy was performed after further workup. Perhaps, if the mass's blood supply had been identified, considerations for preoperative embolization may have been discussed to facilitate a robotic approach. 

## Conclusions

The preoperative planning phase is of utmost importance when tackling mediastinal tumours. It is especially important when coordinating with other services such as otorhinolaryngology. Pathologies vary in presentation, with rare tumours like paragangliomas, a rare neuroendocrine tumour, and thyroid goiters, which are more common. In the presented cases, the later involvement of consultant services may have led to a delay in definitive operation. Moving forward, the utility of volumetric analysis through CT scanning and the consideration of pre-operative embolization for mediastinal tumors in close proximity to the great vessels should be considered to facilitate a successful minimally invasive operation. When all else fails, a reliable and safe operation can be performed via a sternotomy approach, as demonstrated in this case series. The purpose of this study is to emphasize the preoperative planning associated with mediastinal mass excisions and to highlight the varying outcomes associated with rare and common tumors.

## References

[1] Putnam JB (2016). Lung, chest wall, pleura, mediastinum. Sabiston Textbook of Surgery: The Biological Basis of Modern Surgical Practice. 20th ed.

[2] Ahuja J, Strange CD, Agrawal R, Erasmus LT, Truong MT (2023). Approach to imaging of mediastinal masses. Diagnostics (Basel).

[3] Azour L, Moreira AL, Washer SL, Ko JP (2020). Radiologic and pathologic correlation of anterior mediastinal lesions. Mediastinum.

[4] Juanpere S, Cañete N, Ortuño P, Martínez S, Sanchez G, Bernado L (2013). A diagnostic approach to the mediastinal masses. Insights Imaging.

[5] Duwe BV, Sterman DH, Musani AI (2005). Tumors of the mediastinum. Chest.

[6] Chávez Tostado KV, Velázquez-Fernandez D, Chapa M, Pantoja Millán JP, Salazar MS, Herrera MF (2018). Substernal goiter: correlation between grade and surgical approach. Am Surg.

[7] Sormaz İC, Uymaz DS, İşcan AY (2018). The value of preoperative volumetric analysis by computerised tomography of retrosternal goiter to predict the need for an extra-cervical approach. Balkan Med J.

[8] Gaitan E, Nelson NC, Poole GV (1991). Endemic goiter and endemic thyroid disorders. World J Surg.

[9] Doulaptsi M, Karatzanis A, Prokopakis E, Velegrakis S, Loutsidi A, Trachalaki A, Velegrakis G (2019). Substernal goiter: treatment and challenges. Twenty-two years of experience in diagnosis and management of substernal goiters. Auris Nasus Larynx.

[10] Nankee L, Chen H, Schneider DF, Sippel RS, Elfenbein DM (2015). Substernal goiter: when is a sternotomy required?. J Surg Res.

[11] Al-Jehani Y, Saleh W, Al Halees Z, Ashour M (2012). Successful resection of a huge paraganglioma utilizing cardiopulmonary bypass. Asian Cardiovasc Thorac Ann.

[12] Paul S, Jain SH, Gallegos RP, Aranki SF, Bueno R (2007). Functional paraganglioma of the middle mediastinum. Ann Thorac Surg.

[13] Buchanan SN, Radecki KM, Chambers LW (2017). Mediastinal paraganglioma. Ann Thorac Surg.

[14] Wald O, Shapira OM, Murar A, Izhar U (2010). Paraganglioma of the mediastinum: challenges in diagnosis and surgical management. J Cardiothorac Surg.

